# Rising Healthcare Costs and Utilization among Young Adults with Cirrhosis in Ontario: A Population-Based Study

**DOI:** 10.1155/2022/6175913

**Published:** 2022-03-09

**Authors:** Jeffrey B. Ames, Maya Djerboua, Norah A. Terrault, Christopher M. Booth, Jennifer A. Flemming

**Affiliations:** ^1^Departments of Medicine, Oncology, and Public Health Sciences, Queen's University, Kingston, Canada; ^2^IC/ES, Queen's University, Kingston, Canada; ^3^Department of Medicine, University of Southern California, Los Angeles, CA, USA

## Abstract

**Objectives:**

Chronic diseases account for the majority of healthcare spending. Cirrhosis is a chronic disease whose burden is rising, especially in young adults. This study aimed at describing the direct healthcare costs and utilization in young adults with cirrhosis compared to other chronic diseases common to this age group.

**Methods:**

Retrospective population-based study of routinely collected healthcare data from Ontario for the fiscal years 2007–2016 and housed at ICES. Young adults (aged 18–40 years) with cirrhosis, inflammatory bowel disease (IBD), and asthma were identified based on validated case definitions. Total and annual direct healthcare costs and utilization were calculated per individual across multiple healthcare settings and compared based on the type of chronic disease. For cirrhosis, the results were further stratified by etiology and decompensation status.

**Results:**

Total direct healthcare spending from 2007 to 2016 increased by 84% for cirrhosis, 50% for IBD, and 41% for asthma. On a per-patient basis, annual costs were the highest for cirrhosis ($6,581/year) compared to IBD ($5,260/year), and asthma ($2,934/year) driven by acute care in cirrhosis and asthma, and drug costs in IBD. Annual costs were four-fold higher in patients with decompensated versus compensated cirrhosis ($20,651/year vs. $5,280/year). Patients with cirrhosis had greater use of both ICU and mental health services.

**Conclusion:**

Healthcare costs in young adults with cirrhosis are rising and driven by the use of acute care. Strategies to prevent the development of cirrhosis and to coordinate healthcare in this population through the development of chronic disease prevention and management strategies are urgently needed.

## 1. Introduction

Collectively, chronic diseases account for two-thirds of deaths in Canadians, are responsible for 58% of total direct healthcare costs, [1] and, in Ontario, are estimated to account for 55% of total direct and indirect healthcare costs [2]. In response to this, Canada has developed the Chronic Disease Prevention and Management (CDPM) framework, which has been successfully applied to a number of chronic diseases including heart failure [3], diabetes, [4] chronic obstructive pulmonary disease (COPD), [2, 5, 6] and asthma [7]. These programs have successfully reduced hospitalization rates, and improved both quality of life and mortality [5, 8–11].

Cirrhosis refers to an advanced stage of hepatic fibrosis and represents the final common pathway of multiple chronic liver diseases (CLD) with a median survival of <3 years once decompensation occurs [12]. Over the past two decades, the burden of cirrhosis has increased substantially with prevalence reaching almost 1% of the Ontario population in 2016 [13]. Of most concern, however, the highest increase in new diagnoses of cirrhosis has been observed in young adults driven mostly by alcohol-associated disease (ALD) and nonalcoholic fatty liver disease (NAFLD) [14, 15]. Furthermore, in Canadian adults aged 35–64 years, CLD and cirrhosis were the fifth leading causes of death in 2018 behind only cancer, heart disease, accidents, and suicide with mortality rates in this age group increasing by 25% between 2000 and 2018 [16].

Despite these alarming trends and advocacy from the Canadian hepatology community [17], cirrhosis has not been targeted for CDPM strategies [18]. The existing literature on chronic disease programs in cirrhosis has demonstrated improvement in numerous quality of care metrics including frequent patient contact, adherence to guidelines, and care coordination [19–22]. Moreover, these interventions are likely to be cost-effective [23, 24]. To develop such strategies in Canada, understanding the burden of cirrhosis on the healthcare system is an important first step, especially in young adults with cirrhosis, where the disease burden has been shown to be increasing most rapidly. The aims of this study were to describe the direct healthcare costs and healthcare utilization in young adults with cirrhosis and compare this to other chronic conditions that are common in this age demographic (inflammatory bowel diseases [IBD]) for which CDPM strategies already exist (asthma).

## 2. Methods

### 2.1. Study Design and Data Sources

This is a retrospective cohort study utilizing routinely collected administrative healthcare data in the province of Ontario, Canada, and electronically stored at ICES (formerly the Institute of Clinical Evaluative Sciences). ICES is an independent, nonprofit organization for health services research. ICES links administrative healthcare data routinely collected from the single-payer healthcare system called the Ontario Health Insurance Plan (OHIP) with a unique ICES identifier number allowing linkage to a multitude of data sources for use in determining cost and healthcare utilization patterns. Details regarding the databases used are outlined in the Supplementary Material “Appendix 1: ICES Database,” which outlines how this database is created and the types of information found within it. All databases were linked at the individual level and analyzed at ICES Queen's. This study was approved by the Health Sciences Research Ethics Board at Queen's University (DMED 1651–13).

### 2.2. Study Cohort

The study cohort included incident and prevalent cases of cirrhosis [25], IBD [26], and asthma [27] identified using validated case definitions for each condition in individuals between 18 and 40 years of age from April 1, 2007 to March 31, 2017 reflecting fiscal years 2007–2016. The lookback window for capturing prevalent cases continued until the earliest availability for each data source (July 1988 for CIHI-DAD inpatient data, July 1991 for OHIP physician claims) with the first reported instance of cirrhosis, IBD, or asthma representing the date of diagnosis, while incident cases were identified if their initial cirrhosis diagnosis occurred between the study start and end date. For both the IBD and asthma cohorts, we excluded those who had a diagnosis of cirrhosis prior to cohort entry, and similarly, in those with IBD/asthma who were subsequently diagnosed with cirrhosis, we censored their follow-up time at the time of cirrhosis diagnosis. Patients without eligible OHIP coverage were excluded from the study. For a more detailed description of how the IBD and asthma cohorts were created, please refer to Supplementary Material Appendix 2: creation of IBD and asthma cohorts.

### 2.3. Demographics and Descriptors

Age, sex, and date of death were obtained from the RPDB. Socioeconomic status (SES) and urban (>10,000) or rural (<10,000) location were defined using postal codes in the RPDB and described as income quintiles from Statistics Canada. Comorbid illness was described using the Charlson Comorbidity Index [28]. A most responsible etiology of cirrhosis was assigned using a hierarchical algorithm, which incorporates viral serology and ICD coding as previously described [29] and categorized as hepatitis C (HCV), hepatitis B (HBV), autoimmune liver disease (AILD: primary biliary cholangitis, primary sclerosing cholangitis, autoimmune hepatitis), genetic (hereditary hemochromatosis, Wilson's disease), ALD, or NAFLD. Decompensated cirrhosis was identified using a validated algorithm [30]. Individuals with IBD were classified as Crohn's, ulcerative colitis, or indeterminate [26].

### 2.4. Estimation of Direct Healthcare Costs and Healthcare Utilization

#### 2.4.1. Direct Healthcare Costs

Direct healthcare costs refer to those that are directly attributable to healthcare utilization such as physician, nursing, inpatient, and medical testing costs. Given that there is no way to determine from administrative data which costs are related specifically to managing a certain chronic condition, all costs during the study period were evaluated. Cost estimates from physician visits or claims were derived from the fee or amount paid at each encounter according to their respective database (OHIP for physician claims, ODB for drug claims, and HCD for home care services). Patient encounters where the episodes were short or <60 days (such as inpatient hospitalizations, outpatient or same day surgery hospital visits, or emergency department visits) had costs calculated by multiplying the resource intensity weights (RIWs) by the cost per weighted case (CPWC). In addition to the total direct costs derived from the costing algorithm, specific cost categories were also featured and described, including inpatient costs, emergency room costs, subspecialist costs, and prescription drug costs. Costs for physician visits with a gastroenterology (GI) subspecialty or respirology subspeciality were calculated for subspecialty cost category. Costs were reported in Canadian dollars (CAD), prorated where applicable, and adjusted for inflation to 2017.

#### 2.4.2. Healthcare Utilization

Healthcare utilization was captured for each disease cohort through the number of unique inpatient hospitalizations, emergency department visits, and physician subspecialty and primary care visits per fiscal year during the study period. Outpatient mental health service utilization was identified when the billing physician specialty code was defined as “Psychiatry.” ER visits and inpatient admissions for chronic mental health were identified based on a validated definition in ICES [31]. The proportion of admissions for each fiscal year that were 30-day readmissions (where the admission date was less than 30 days after the discharge date for the initial hospitalization) were also reported.

### 2.5. Statistical Analyses

#### 2.5.1. Demographics

Cross-tabulations were used to describe demographics of each cohort and the frequency and proportion of patients within specified categorical variables, while means and standard deviations were used for numeric variables.

#### 2.5.2. Healthcare Costs

The sum and mean total direct costs per patient for each fiscal year from 2007 to 2016 were calculated for each disease cohort using a costing algorithm deployed through a SAS macrodeveloped by and available at ICES [32]. Costs for the cirrhosis cohort were further stratified by cirrhosis etiology and cirrhosis decompensation status. Given drug costs are not captured universally for the cohort, sensitivity analyses were performed by excluding drug costs. Upon establishing the total and subcategory costs per patient, patients in the cirrhosis cohort were organized into deciles based on the distribution of total cost for the study period. Within each total cost decile, the proportion of the costs according to each cost subcategory (subspecialist costs, inpatients costs, ER costs, etc.) were calculated out of the total costs.

#### 2.5.3. Healthcare Resource Utilization and Cost Usage Patterns

The number of healthcare utilization services was expressed as percentiles stratified by the type of chronic disease and also stratified by cirrhosis etiology and decompensation status. Cost usage patterns for individuals with cirrhosis over the study period were evaluated for each patient's index fiscal year (i.e., the patient's diagnosis year for incident cases or the first study year (i.e., year 2000) for prevalent cases). Based on previous costing studies using administrative healthcare data [33], patients were categorized into groups based on their usage of the total cost for the entire cohort in the index fiscal year. Patients whose total cost for the index fiscal year was <50th percentile of the total cohort cost were categorized as low-cost users, patients within the 50–89th percentile were categorized as moderate-cost users, patients above the 90th percentile were categorized as high-cost users, and patients above the 95th percentile were categorized as very high-cost users. The same cost usage categorization was applied to the subsequent three fiscal years. Patients were stratified according to their cost usage category in their index year and their number of follow-up years, and the proportion of patients who remained in the same cost usage category or moved to a higher or lower cost usage category within the 3-year follow-up period was calculated to track the patterns and transitions of cost usage in young adults with cirrhosis.

All analyses were performed using SAS Enterprise Guide version 7.1 (SAS Institute, Cary, NC, USA).

## 3. Results

### 3.1. Demographics of Young Adults with Cirrhosis, IBD, and Asthma

**Figure 1:**
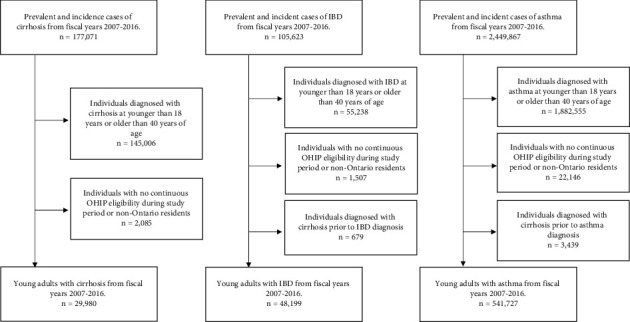
Inclusion and exclusion criteria for the creation of young cirrhosis, IBD, and asthma disease cohorts.

The development of the study cohort is outlined in Figure 1, and demographics of the cohorts are shown in Table 1. Overall, 29,980 young adults with cirrhosis, 48,199 with IBD, and 541,727 with asthma were included. The median age at diagnosis was 33 years for cirrhosis, 29 years for IBD, and 30 years for asthma. Males made a higher proportion in the cirrhosis cohort (57%), while a higher female proportion (62%) was observed for asthma with even distribution in those with IBD (47% male). Young adults with cirrhosis had higher comorbidity than those with IBD and asthma. For cirrhosis, the majority had NAFLD as the etiology (60%) followed by ALD (16%) and HCV (11%), and 3,493 (12%) had decompensated disease at some point during the study period. Drug coverage data in the ODB database were available for 50% of individuals with cirrhosis, 48% with IBD, and 45% with asthma.

### 3.2. Direct Healthcare Costs in Young Adults with Cirrhosis, IBD, and Asthma

**Figure 2:**
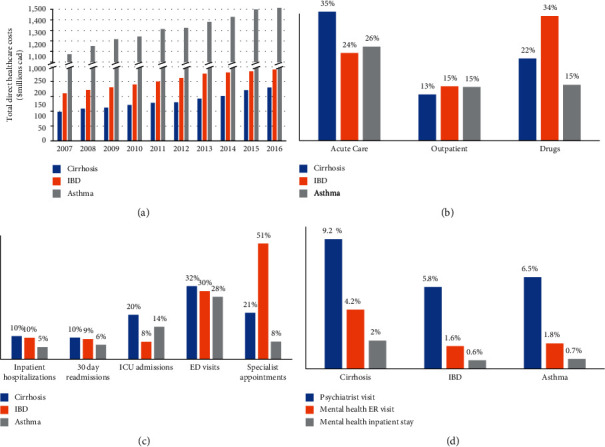
(a) Overall direct healthcare costs for young adults with chronic disease in Ontario (2007–2016). (b) Proportion of annual direct healthcare costs in young adults with chronic disease in Ontario 2016 stratified by resource type. (c) Proportion of young adults with chronic disease utilizing healthcare resources in 2016 in Ontario. (d) Proportion of young adults with chronic disease utilizing mental health resources in 2016 in Ontario.

The total annual direct healthcare costs from 2007 to 2016 stratified by the type of chronic disease are shown in Figure 2(a). For young adults with cirrhosis, the total direct heathcare cost in 2007 was $97.7 million (*M*) and rose to $179.6 M in 2016 representing an 84% increase over the 10-year study. Comparatively, 10-year increases of 50% and 41% were seen for the IBD and asthma cohorts, respectively. The mean annual per-patient direct healthcare costs for the calendar year 2016 stratified by chronic disease and type of resource (inpatient, ER, drug, and subspecialist) are shown in Table 2. On an average per-patient basis, overall costs in 2016 were highest in young adults with cirrhosis ($6,581/year) compared to IBD ($5,260/year) and asthma ($2,934/year), which persisted after excluding drug costs (Supplemental Table 1). When stratified by the decompensation status, annual per-patient costs were over four-fold higher for decompensated cirrhosis compared to compensated cirrhosis (decompensated: $20,651/year vs. compensated: $5,280/year). Costs stratified by resource type in 2016 (Figure 2(b)) demonstrate the highest proportion of direct costs in young adults with cirrhosis came from acute care (35%), followed by drug costs (22%) and outpatient care (13%) and were similar to patients with asthma (acute care 26%, drugs 15%, and outpatient care 15%). Conversely, drugs accounted for the majority of direct costs in patients with IBD (34%).

### 3.3. Annual Healthcare Utilization in Young Adults with Cirrhosis, IBD, and Asthma

Healthcare utilization in 2016 stratified by the type of chronic disease is shown in Figure 2(c). Hospital admissions were higher for cirrhosis (10%) and IBD (9%) compared to asthma (6%), with individuals with cirrhosis more frequently admitted to an ICU (20%) compared to IBD (8%) and asthma (14%). The proportion with a 30-day readmission (cirrhosis 10%; IBD 9%; asthma 6%) was similar between all conditions and one-third of all young adults with chronic disease had an ER visit. Young adults with IBD more commonly sought outpatient subspecialist care (GI visit: 51%) compared to cirrhosis (GI visit: 21%) and asthma (respirologist visit: 8%) with primary care usage comparable across conditions (cirrhosis: 81%; IBD: 84%; asthma: 84%). The use of mental health services was more frequent in young adults with cirrhosis (Figure 2(d)).

### 3.4. Healthcare Costs and Utilization in Young Adults with Cirrhosis Stratified by Etiology

**Figure 3:**
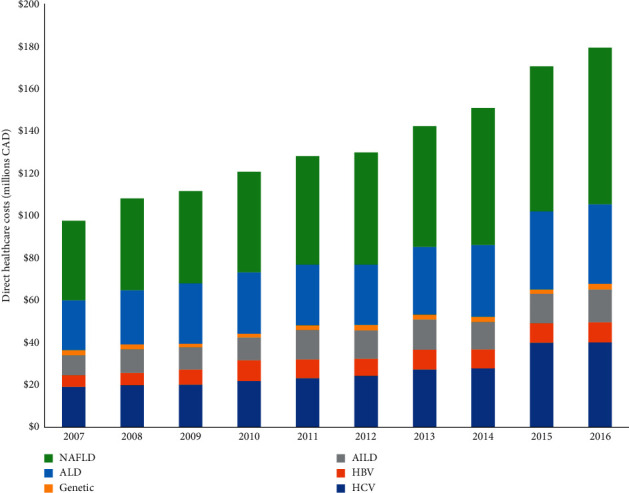
Overall direct healthcare costs in young adults with cirrhosis in Ontario 2007-2016. NAFLD: nonalcoholic fatty liver disease; HCV: hepatitis C; ALD: alcohol-associated liver disease; HBV: hepatitis B; AILD: autoimmune liver disease.

**Figure 4:**
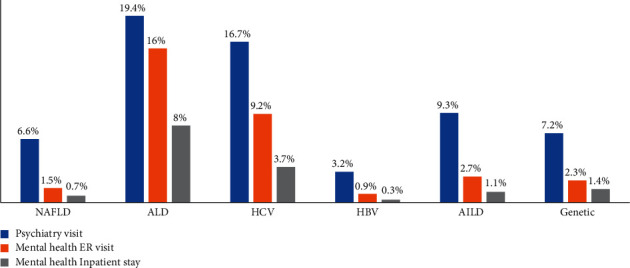
Proportion of young adults with cirrhosis utilizing mental health services in Ontario in 2016. NAFLD: nonalcoholic fatty liver disease; ALD: alcohol-related liver disease; HCV: hepatitis C; HBV: hepatitis B, AILD: autoimmune liver disease.

Increases in total direct healthcare expenditures from 2007 to 2016 stratified by cirrhosis etiology (Figure 3) were the highest for young adults with cirrhosis secondary to HCV (+109%) followed by NAFLD (+97%), HBV (+73%), AILD (+65%), ALD (+59%), and genetic causes (+17%). Healthcare costs and utilization in 2016 stratified by etiology are shown in Table 3. The mean direct per-patient healthcare costs were the highest in those with HCV cirrhosis ($14,040/year), followed by AILD ($12,781/year) and genetic etiologies ($12,410/year) and lowest in those with NAFLD ($4,329/year). However, due to the burden of disease, the total annual costs were the highest for the care of young adults with NAFLD cirrhosis ($74.1 M/year), followed by HCV ($40.2 M/year), and ALD ($37.6 M/year). For acute healthcare utilization, inpatient admissions and ER visits were most frequent in young adults with ALD (hospitalization: 21%; ER: 50%) and AILD cirrhosis (hospitalization: 21%; ER: 42%), and lowest in NAFLD (hospitalization: 7%; ER: 29%) and HBV (hospitalization: 5%; ER: 17%). Finally, the use of inpatient and outpatient mental health services was the highest in those with ALD and HCV cirrhosis and lowest in HBV and NAFLD (Figure 4).

### 3.5. Healthcare Costs by Decile in Young Adults with Cirrhosis

**Figure 5:**
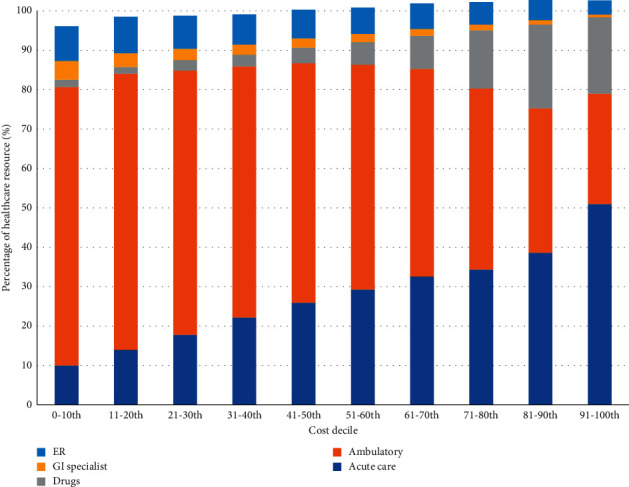
Healthcare resource utilization in young adults with cirrhosis by cost decile in 2016. ER: emergency room; GI: gastroenterology.

**Figure 6:**
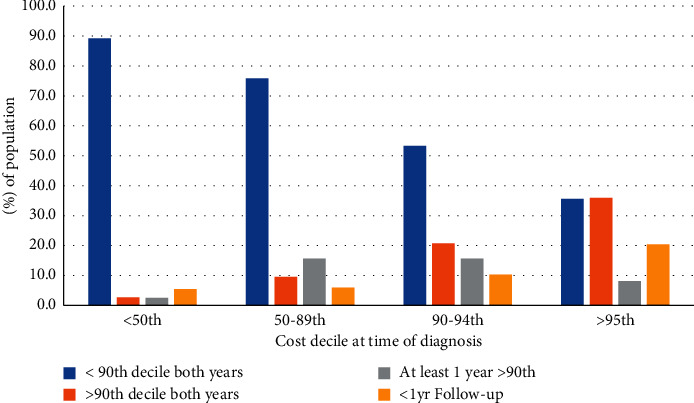
Total cost percentile transition patterns within 3 years of initial diagnosis in young adults with cirrhosis.

Healthcare costs in young adults with cirrhosis from 2007 to 2016 stratified by cost decile (Figure 5) demonstrate an increase in the proportion of costs arising from acute care resources and drug costs with increasing cost deciles and a corresponding reduction in costs related to ambulatory care. Over 60% of direct costs in the top 1% of young adults with cirrhosis are spent on acute care resources compared to only 10% at the 0–10th cost decile. Finally, there is longitudinal stability of users within each cost bracket with 33% of high-cost users and 42% of very-high-cost users remaining in either the high- or very-high-cost bracket after 3 years of follow-up (Figure 6).

## 4. Discussion

Over the past decade, direct healthcare costs associated with caring for young adults with chronic diseases have increased substantially in Ontario, with the highest annual per-patient healthcare expenditures seen in individuals with cirrhosis compared to IBD and asthma. Direct costs in young adults with cirrhosis, especially those in the top decile of spending, are driven largely by the use of acute care resources, with the average annual per-patient cost in 2016 exceeding $20,000/year in those with decompensated disease. Given the high burden of disease, overall direct healthcare spending is the highest for young adults with NAFLD; however, on an individual basis, costs are greatest for those with HCV. The proportion utilizing acute care and mental health resources are the highest in young adults with ALD, and when admitted to hospital, ∼20% require ICU level care. These data suggest that the development of CDPM programs focusing on strategies aimed at decreasing the need for acute healthcare resources and the prevention of decompensation could result in decreased direct healthcare costs and utilization.

To our knowledge, no previous studies have evaluated the direct healthcare costs and utilization in young adults with cirrhosis nor compared these results to cohorts of young adults with other chronic illnesses. However, our data are consistent with studies performed in the overall adult cirrhosis population. Data from the United States have demonstrated a 30% increase in direct healthcare costs from 2008 to 2014 for hospitalization only claims in those with cirrhosis compared to 4% in those without cirrhosis [34]. Similarly, in another US study comparing healthcare utilization in adults with cirrhosis (median age 57) to other chronic diseases, individuals with cirrhosis were found to have higher rates of hospitalization, longer lengths of stay, and higher inpatient mortality [35]. Therefore, our results suggest that these same trends extend to the young adult population where costs and utilization in individuals with cirrhosis exceed those of other chronic conditions. It is also worth noting that in this cohort of young adults with cirrhosis, over 90% had no other comorbid illness as identified by the Charlson Comorbidity Index, and therefore, costs are almost exclusively related to complications of liver disease, which may not be true in an older population with more prevalent comorbid conditions, which also require healthcare utilization.

When examining differences in healthcare costs between the three chronic disease cohorts, overall costs rose the highest in young adults with cirrhosis. This may be explained by several factors. First, the incidence of cirrhosis in young adults has increased substantially in Ontario over the past two decades [15]. Although incidence has been shown to be increasing in children with IBD [36] and asthma [37] in Ontario, rates in the adult IBD population in Ontario have not been described, the incidence of IBD in adults in Nova Scotia has actually been declining [38], while adult asthma incidence rates have been stable [37]. Secondly, new direct acting antiviral therapies for HCV have been available since 2016, which is also leading to higher costs. Finally, inpatient costs in decompensated cirrhosis are ∼4 times higher than that of patients with IBD or asthma. This again highlights the need to prevent hepatic decompensation, which in turn should lower overall costs substantially.

Acute care resource use was highest among young adults with cirrhosis, particularly those with decompensated disease, and therefore, targeting acute care utilization is an obvious strategy to reduce healthcare spending in this population. Previous studies in patients with cirrhosis have shown that the use of decision support tools [12] and early follow-up with comprehensive specialized clinics [23, 39] are associated with reductions in 30-day readmission rates, ER visits, 60-day mortality, and improved cost-effectiveness. The use of early palliative care has been associated with reductions in resource utilization in those with decompensated disease [40]. Therefore, given the data showing the importance of specialist clinic involvement (GI, hepatology, and palliative care) [16, 20], understanding barriers to referral and timely access to specialty clinics is important. Indeed, in our study, only 20% of young adults with cirrhosis had a visit with a GI/hepatologist in the 2016 calendar year; however, it is unclear whether this relates to barriers in access to care, low rates of referral, management occurring at the primary care level, or patient-related factors. Also, given the high use of mental health services and comorbid mental health conditions in those with ALD and HCV, facilitating access to mental health professionals will also be essential and may be achieved by the creation of joint hepatology, addiction, and psychiatric clinics [41].

Ultimately, early identification of NAFLD, HCV, and ALD with a goal to prevent cirrhosis and decompensation should be key priorities for public health. Modelling studies suggest that without intervention, the prevalence of NAFLD and NAFLD cirrhosis will continue to increase in Canada and elsewhere over the next several decades [14, 42, 43]. This represents a tremendous opportunity to potentially decrease the burden of cirrhosis and progression to decompensation with early identification, lifestyle interventions, consideration for therapeutics, and bariatric surgery [44] with screening essential at the level of primary care. Screening for NAFLD has recently been shown to be cost-effective in those with diabetes [45], and the European Association for the Study of Liver Disease endorses screening high-risk groups (age >50, T2DM, metabolic syndrome) [46, 47]. The specifics on how to roll out such an effort have yet to be elucidated; however, recent data using artificial intelligence and serum-based fibrosis markers in electronic health records are a promising first step. Of note, the majority of biochemical screening tests for fibrosis assessment (i.e., FIB-4, NAFLD fibrosis score) were derived in populations of much older individuals, and therefore, validation of these tools in younger populations is needed [48]. The use of direct acting antiviral therapy in those with HCV is associated with decreases in both liver-related and all-cause mortality [49–51] and therefore identifying and treating HCV in young adults before cirrhosis development is paramount. Finally, given the expected increase in ALD cirrhosis over the next 20 years in younger generations [52], screening for AUD and ALD needs to be prioritized along with the ongoing development of therapeutic options to manage this resource-intensive patient population.

Limitations of this study include those inherent of a retrospective cohort study using large administrative databases including selection and misclassification bias. Drug costs are underrepresented as universal drug coverage is not offered in Ontario and only ∼50% of the study cohort was covered under ODB, which captures medication costs. Lastly, given the heterogeneity of cirrhosis epidemiology worldwide, external validity of these data outside of Canada needs to be considered.

This study highlights the high costs and healthcare resource use in young adults with cirrhosis, which is highly dependent on acute care resources when compared to other similarly aged chronic disease cohorts, demonstrating an unequivocal need for improved chronic disease prevention and management in this heterogenous population. Important areas to address include the increasing burden of NAFLD and a need for improved quality and coordination of care across resource settings. Further research is needed to investigate cost-effective interventions at these various points with the goal of developing a robust CDPM framework for chronic liver disease.

## Figures and Tables

**Table 1:** Demographic and disease characteristics of young adults with cirrhosis, IBD, and asthma in Ontario 2007–2016.

	Cirrhosis (*n* = 29,980)	IBD (*n* = 48,199)	Asthma (*n* = 541,727)
Age, median years (IQR)	33 [28–37]	29 [24–35]	30 [24–35]
Male sex, *n* (%)	17,132 (57)	22,455 [47]	207,145 [38]
Cirrhosis etiology, *n* (%)			
NAFLD	17,949 (60)	—	—
ALD	4,648 [16]	—	—
HCV	3,320 [11]	—	—
HBV	2,424 [8]	—	—
AILD	1,394 [5]	—	—
Genetic	245 [1]	—	—
IBD subtype, *n* (%)			
Crohn's disease	—	23,008 [48]	—
Ulcerative colitis	—	23,442 [49]	—
Indeterminate colitis	—	1,749 [4]	—
Income quintile, *n* (%)			
1—lowest	7,770 [26]	8,467 [18]	127,659 [24]
2	6,406 [21]	9,509 [20]	113,104 [21]
3	5,843 [20]	9,693 [20]	106,340 [20]
4	5,358 [18]	10,498 [22]	100,813 [19]
5—highest	4,371 [15]	9,582 [20]	88,158 [17]
Missing	232 [1]	450 [1]	5,653 [1]
Rurality, *n* (%)			
>10,000 (urban)	27,228 (91)	42,438 (88)	483,158 (90)
≤10,000 (rural)	2,718 [9]	5,619 [12]	56,777 [10]
Missing	34 (0.1)	142 (0.3)	1,792 (0.3)
CCI, *n* (%)			
0	27,821 (93)	47,449 (98)	531,292 (98)
1	955 [3]	454 [1]	6,595 [1]
2–3	780 [3]	242 (0.5)	3,010 [1]
≥4	415 [1]	54 (0.1)	830 (0.2)
ODB utilization, *n* (%)	15,089 [50]	22,922 [48]	243,365 [45]

IBD: inflammatory bowel disease; IQR: interquartile range; UC: ulcerative colitis; NAFLD: nonalcoholic fatty liver disease; ALD: alcohol-related liver disease; HCV: hepatitis C; HBV: hepatitis B; AI: autoimmune liver disease; CCI: Charlson Comorbidity Index.

**Table 2:** Direct per-patient healthcare costs in young adults with cirrhosis, IBD, and asthma in Ontario in 2016.

	Overall cirrhosis *n* = 27,305	Compensated cirrhosis *n* = 24,995	Decompensated cirrhosis *n* = 2,310	IBD *n* = 45,719	Asthma *n* = 516,135
Inpatient costs					
Per-patient Mean CAD (sd)	$2,029 ($14,183)	$1,261 ($10,283)	$10,337 ($34,035)	$1,060 ($6,715)	$578 ($5,475)
Total CAD	$55,400,782	$31,520,276	$23,880,506	$48,470,341	$298,156,358
ER cost					
Per-patient Mean CAD (sd)	$280 ($1,121)	$245 ($1,077)	$660 ($1,464)	$198 ($616)	$156 ($509)
Total CAD	$7,651,738	$6,126,849	$1,524,889	$9,072,340	$80,601,031
Subspecialist costs^*∗*^					
Per-patient Mean CAD (sd)	$65 ($254)	$45 ($176)	$282 ($613)	$138 ($248)	$20 ($130)
Total CAD	$1,794,076	$1,141,742	$652,334	$6,329,228	$10,325,815
Drug costs					
Per-patient Mean CAD (sd)	$1,471 ($9,296)	$1,349 ($8,510)	$2,805 ($15,364)	$1,563 ($6,762)	$430 ($3,711)
Total CAD	$40,185,123	$33,704,164	$6,480,959	$71,470,193	$221,796,789
Total direct costs					
Per-patient Mean CAD (sd)	$6,581 ($ 22,276)	$5,280 ($17,967)	$20,651 ($46,438)	$5,260 ($13,115)	$2,934 ($9,906)
Total CAD	$179,671,525	$131,967,823	$47,703,702	$240,463,283	$1,514,158,577

IBD: inflammatory bowel disease; CAD: Canadian dollars; sd: standard deviation. ^*∗*^For cirrhosis and IBD, subspecialist is gastroenterology; for asthma subspecialist is respirology.

**Table 3:** Direct healthcare costs and utilization of young adults with cirrhosis in Ontario (2016).

Direct costs	NAFLD *n* = 17,106	ALD *n* = 3,591	HCV *n* = 2,863	HBV *n* = 2,312	AILD *n* = 1,211	Genetic *n* = 222
Inpatient						
Per-patient mean CAD (sd)	$1,167 ($10,419)	$4,555 ($19,404)	$3,201 ($20,390)	$904 ($12,327)	$5,711 ($22,413)	$4,107 ($18,420)
Total CAD	$19,961,196	$16,357,310	$9,162,395	$2,091,184	$6,916,866	$911,831
Emergency room						
Per-patient mean CAD (sd)	$178 ($555)	$715 ($2,531)	$432 ($1,105)	$93 ($332)	$420 ($925)	$324 ($957)
Total CAD	$3,046,894	$2,569,248	$1,239,093	$215,067	$509,383	$72,053
Subspecialist						
Per-patient mean CAD (sd)	$37 ($179)	$100 ($367)	$77 ($262)	$77 ($173)	$299 ($540)	$108 ($345)
Total CAD	$646,619	$361,728	$220,723	$178,082	$362,842	$24,082
Drug						
Per-patient mean CAD (sd)	$768 ($8,588)	$1,146 ($3,021)	$6,366 ($18,603)	$1,162 ($3,457)	$1,279 ($ 4,472)	$2,102 ($ 7,039)
Total CAD	$13,142,439	$4,117,969	$18,220,342	$2,688,140	$1,549,503	$466,730
Overall						
Per-patient mean CAD (sd)	$4,330 ($18,337)	$10,475 ($26,721)	$14,041 ($31,468)	$4,146 ($16,627)	$12,782 ($31,520)	$12,411 ($29,329)
Total CAD	$74,050,835	$37,616,964	$40,184,953	$9,585,003	$15,478,568	$2,755,202
Utilization						
Hospitalization, *n* (%)	1,237 (7.2)	750 (20.9)	398 (9.5)	116 (5.0)	256 (21.1)	33 (14.9)
Length of stay, med (IQR)	3 [2–8]	6 [3–16]	6 [3–14]	3 [2–7]	7 [3–21]	5 [3–20]
ICU admission, *n* (%)	308 (17.2)	305 (20.6)	159 (22.5)	34 (11.2)	109 (21.4)	14 (19.5)
30-day readmission, *n* (%)	135 (7.5)	164 (11.1)	67 (9.5)	10 (6.6)	67 (13.1)	8 (11.1)
ER visit, *n* (%)	4,893 (28.6)	1,786 (49.7)	1,180 (41.2)	400 (17.3)	505 (41.7)	71 (32.0)
GI visit, *n* (%)	2,240 (13.1)	767 (21.4)	738 (25.8)	968 (41.9)	806 (66.6)	80 (36.0)
Primary care visit, *n* (%)	13,908 (81.3)	2,927 (81.5)	2,422 (84.6)	1,828 (79.1)	1.026 (84.7)	171 (77.0)

NAFLD: nonalcoholic fatty liver disease; ALD: alcohol-related liver disease; HCV: hepatitis C; HBV: hepatitis B; AILD: autoimmune liver disease; CAD: Canadian dollars; sd: standard deviation; IQR: interquartile range; med: median; ICU: intensive care unit; ER: emergency room; GI: gastroenterologist.

## Data Availability

The dataset from this study is held securely in coded form at ICES. While legal data sharing agreements between ICES and data providers (e.g., healthcare organizations and government) prohibit ICES from making the dataset publicly available, access may be granted to those who meet prespecified criteria for confidential access, available at http://www.ices.on.ca/DAS (e-mail: das@ices.on.ca). The full dataset creation plan and underlying analytic code are available from the authors upon request, understanding that the computer programs may rely upon coding templates or macros that are unique to ICES and are therefore either inaccessible or may require modification.

## Additional Points

*Highlights*. (i) The incidence and overall burden of cirrhosis in the young population is increasing. (ii) Direct healthcare costs related to the young cirrhosis population and patterns of healthcare resource use are not well documented. (iii) In the past decade, direct healthcare costs have increased proportionally more in the young cirrhosis population compared to other similarly matched chronic disease populations. (iv) Nonalcoholic fatty liver disease (NAFLD) accounts for the largest proportion of healthcare spending within this population. (v) Costs are driven predominantly by acute care resource use with patterns of use strongly affected by decompensation status and cost decile.

## Supplementary Materials

Supplementary Materials Appendix 1: ICES Database. Approximately 14 million people reside in Ontario and receive universal healthcare coverage for all physician services under a single-payer system called the Ontario Health Insurance Plan (OHIP). Health administrative data are collected routinely from individuals who are eligible for OHIP. All Ontario residents with an eligible or valid OHIP healthcare number are given a unique ICES identifier number that allows direct linkage to a multitude of data sources situated at ICES. Data sources at ICES utilized to determine cost and healthcare utilization patterns in this study included the OHIP physician claims database, which includes information on all healthcare services billed under OHIP from 1991 onwards, Canadian Institute for Health Information Discharge Abstract Database (CIHI-DAD), which includes all inpatient hospital records from 1988 onwards, National Ambulatory Care Recording System (NACRS), which contains all emergency department and outpatient clinic information from 2000 onwards. The Ontario Drug Benefit (ODB) database provides drug and pharmaceutical cost coverage for Ontarians over the age of 65 years or for those under 65 years who are not covered by a private drug insurance plan. The Complex Continuing Care Reporting Care System (CCRS) provides information on facility-based continuing care from 1996 onwards, and the Home Care Database (HCD), which includes information on home care services from 2005 onwards. The Registered Persons Database (RPDB) is an additional data repository with demographic information such as sex, birth date, postal code, and vital status for all Ontario residents with a valid OHIP number. All databases were linked at the individual level and analyzed at ICES Queen's. Appendix 2: creation of IBD and asthma cohorts. Prevalent and incident cases of IBD diagnosed between 18 and 40 years of age were extrapolated from the Ontario Crohn's and Colitis Cohort database, which uses a case definition of Crohn's disease or ulcerative colitis based on at least five recorded inpatient, emergency department, or outpatient visits within four years with a diagnostic code for IBD (ICD-9: 555, 556; ICD-10: K50, K51) and categorized as Crohn's disease, ulcerative colitis (UC), or indeterminate [26]. Prevalent and incident cases of asthma diagnosed between 18 and 40 years of age were derived from the Ontario Asthma Cohort, which captures cases using a definition wherein individuals must have one inpatient hospitalization or two outpatient visits with an asthma diagnosis (ICD-9: 493; ICD-10: J45, J46) [27].
