# Supplementary material for: Rising Healthcare Costs and Utilization among Young Adults with Cirrhosis in Ontario: A Population-Based Study
Source: Can J Gastroenterol Hepatol. 2022 Mar 9;2022:6175913. doi: 10.1155/2022/6175913 (PMC8926479; doi:10.1155/2022/6175913)
Supplement: Supplementary Materials — Appendix 1: ICES Database. Approximately 14 million people reside in Ontario and receive universal healthcare coverage for all physician services under a single-payer system called the Ontario Health Insurance Plan (OHIP). Health administrative data are collected routinely from individuals who are eligible for OHIP. All Ontario residents with an eligible or valid OHIP healthcare number are given a unique ICES identifier number that allows direct linkage to a multitude of data sources situated at ICES. Data sources at ICES utilized to determine cost and healthcare utilization patterns in this study included the OHIP physician claims database, which includes information on all healthcare services billed under OHIP from 1991 onwards, Canadian Institute for Health Information Discharge Abstract Database (CIHI-DAD), which includes all inpatient hospital records from 1988 onwards, National Ambulatory Care Recording System (NACRS), which contains all emergency department and outpatient clinic information from 2000 onwards. The Ontario Drug Benefit (ODB) database provides drug and pharmaceutical cost coverage for Ontarians over the age of 65 years or for those under 65 years who are not covered by a private drug insurance plan. The Complex Continuing Care Reporting Care System (CCRS) provides information on facility-based continuing care from 1996 onwards, and the Home Care Database (HCD), which includes information on home care services from 2005 onwards. The Registered Persons Database (RPDB) is an additional data repository with demographic information such as sex, birth date, postal code, and vital status for all Ontario residents with a valid OHIP number. All databases were linked at the individual level and analyzed at ICES Queen's. Appendix 2: creation of IBD and asthma cohorts. Prevalent and incident cases of IBD diagnosed between 18 and 40 years of age were extrapolated from the Ontario Crohn's and Colitis Cohort database, which uses a case definition of Crohn's di [file 6175913.f1.zip › 6175913.f1/Supplemental table.docx]

| **Supplemental Table 1:** Direct healthcare costs excluding drug costs in young adults with chronic disease in Ontario 2016. | | | |
| --- | --- | --- | --- |
|  | Cirrhosis  n = 27,305 | IBD  n = 45,719 | Asthma  n = 516,135 |
| Mean cost per patient | $5,109 | $3,696 | $2,504 |
| Total costs | $139,486,402 | $168,993,090 | $1,292,361,788 |
